# Seven-Year Longitudinal Study: Clinical Evaluation of Knee Osteoarthritic Patients Treated with Mesenchymal Stem Cells

**DOI:** 10.3390/jcm13133861

**Published:** 2024-06-30

**Authors:** Dusko Spasovski, Vesna Spasovski, Zoran Bascarevic, Maja Stojiljkovic, Marina Andjelkovic, Sonja Pavlovic

**Affiliations:** 1 Institute for Orthopedics Banjica, University of Belgrade, 11000 Belgrade, Serbia; duskosp@gmail.com (D.S.); zoran.bascarevic@gmail.com (Z.B.); 2 School of Medicine, University of Belgrade, 11000 Belgrade, Serbia; 3 Institute of Molecular Genetics and Genetic Engineering, University of Belgrade, 11000 Belgrade, Serbia; maja.stojiljkovic@imgge.bg.ac.rs (M.S.); marina.andjelkovic@imgge.bg.ac.rs (M.A.); sonja.pavlovic99@gmail.com (S.P.)

**Keywords:** stem cell therapy, osteoarthritis, mesenchymal stem cells, long term follow-up

## Abstract

**Background/Objectives**: Numerous studies have demonstrated the safety and efficacy of intraarticular stem cell injections for treating osteoarthritic knee joints, reporting symptom reduction and pain relief within a few months of treatment. Here, we report the results of a 7-year follow-up after a single intraarticular injection of 0.5–1 × 10^7^ autologous adipose tissue-derived mesenchymal stem cells in patients with OA (Kellgren-Lawrence grade 2 to 4). **Methods**: Nine patients were treated, and two patients had bilateral disease. Patients were evaluated clinically and radiologically using X-ray and MRI. A comprehensive statistical analysis was undertaken to evaluate the obtained results. **Results**: All clinical scores and range of motion significantly improved within the first six months after injection. At the 18-month time point, a significant improvement in cartilage structure was observed on MRI while X-ray showed no changes in subchondral bone of distal femur and proximal tibia. At the 60-month time point, the clinical scores were still improved compared to baseline, except for the range of motion, which decreased almost back to the baseline level. At 84 months, the clinical scores decreased significantly toward the baseline level, but the MRI structural characteristics of cartilage still remained significantly better than those measured at baseline. **Conclusions**: Adipose tissue-derived stem cell therapy has substantial long-term clinical effects on patients with knee osteoarthritis.

## 1. Introduction

Osteoarthritis (OA) is a painful condition in which progressive destruction of hyaline articular cartilage is usually associated with a limited range of motion and reduced quality of life. It is predominant in females and usually coincides with older age and overuse of joints in athletes or due to obesity [1]. In severe cases, OA leads to substantial disability even in younger people, which can have an impact on work ability [2]. Current medical treatments are mostly ineffective and rather expensive; thus, OA has an enormous effect on people’s quality of life, and it is also a burden on healthcare systems and society.

Although the underlying mechanism is quite well understood, the exact cause of OA is still unknown. In that setting, only symptomatic therapy is available. There are a variety of therapeutic approaches, ranging from various injectable lubricants [3] to minimally invasive operative treatments such as arthroscopic surgery [4,5], invasive surgical methods, and knee replacement surgery [6,7]. Biological therapies, including hyaluronic acid [8], platelet-rich plasma (PRP) [9], and adipose tissue aspirate [10,11,12], are also available and widely used. There are methods that combine surgery and biological therapy, such as the microfracture technique [13]. In this procedure, abrasion of the joint is performed during arthroscopic surgery, allowing entry of cells and growth factors from the underlying bone marrow into the affected joint, triggering regenerative processes and healing of the chondral defect [14]. Although biological therapy modalities lead to the relief of symptoms, their outcome is temporary, lasting from several weeks to several months, and most importantly, they do not counteract the progression of the disease. 

The use of propagated mesenchymal stem cells (MSCs) in the treatment of OA has increased therapeutic efficacy. MSCs originating from bone marrow or adipose tissue were used in most of these studies, and their safety and effectiveness were first examined. Good tolerance of a single injection of stem cells and improvement in clinical parameters during the first weeks and months after the application were reported in the first studies. Clinical efficacy has been reported with lower doses of MSCs (ranging from 2 × 10^6^ to 5 × 10^7^) [2,15,16,17,18,19,20], suggesting that efficacy relies on various parameters, including the intrinsic features of stem cells and their interaction with the inflammatory background of the host niche. Nevertheless, injection of 0.5–1 × 10^8^ stem cells per joint led to improvement in cartilage quality [21,22,23], probably through the activation of the internal regenerative potential of the surrounding tissue. 

Assessment of clinical, X-ray, and MRI findings over longer periods is necessary for the estimation of the effects of therapy and the clinical use of stem cells for treating OA. Here, we present the 7-year follow-up outcomes in a cohort study of nine patients (eleven knees were examined, because two patients had bilateral disease) treated with a single intra-articular injection of 0.5–1 × 10^7^ autologous adipose-derived mesenchymal stem cells (AMSCs) [18].

## 2. Material and Methods

### 2.1. Study Design

The study included patients with knee osteoarthritis diagnosed at the Institute for Orthopaedics “Banjica” during the recruitment period (from April till October 2015). The original research was carried out in compliance with the Helsinki Declaration. It was approved by the Serbian Ministry of Health (License No. 500-01-01106/2014-03) and the Ethical Committee of the Institute for Orthopaedic Surgery “Banjica” under the number I-67/9 (2 June 2015). Informed Consent was obtained from all participants.

The study relied on the following inclusion and exclusion criteria:

Inclusion criteria:

- intensity of symptoms (Knee Society score < 60)
- duration of symptoms > 3 months
- refractory to nonoperative treatment
- age between 18–80 years

Exclusion criteria:

- history or signs of oncologic disease
- systemic metabolic disease (diabetes, thyroid diseases etc.)

Nine patients (11 knees, since two patients were treated bilaterally) fulfilled all the criteria and were included in the study. They were clinically examined and scored at 3 months, 6 months, 12 months, and 18 months follow-up time points, with MRI analysis done at 18 months time points, and these results were published [18].

Patients were further examined at the 5 year (60 months) and 7 year (84 months) time points, and these results are presented here. One patient had to be excluded from the study due to developing a systemic disease diagnosed between the 18-months and 60-month follow-up period. The remaining 8 patients (a total of 10 knees) were clinically and radiographically evaluated. At the 84 months time point, all 8 patients were evaluated clinically, and for MRI analysis, one patient was unavailable due to total knee replacement surgery, and another patient refused to undergo MRI; therefore, 8 knees were evaluated using MRI.

During the follow-up period, patients were allowed to engage in standard daily activities. Three patients reported occasional use of oral analgesics only after increased activity events. Two patients received a series of physiotherapy during the recovery period. 

### 2.2. Tissue Sampling, Isolation and Propagation of AD-MSCs

Sampling involved excising 5 mL of subcutaneous fat tissue via a small incision in the superficial abdominal region under local anesthesia. The sample was left overnight at room temperature. Following repeated washing in 1xPBS solution, the tissue underwent treatment with 0.1% Collagenase (Sigma Aldrich, St. Louis, MO, USA) until it was completely dissolved. Autologous serum was prepared by centrifuging whole blood (without anticoagulants) at 1300 rcf for 10 min and filtering through a 0.22 µm filter. The Collagenase solution was neutralized using a Growth medium (DMEM GlutaMAX™ 1 g/L glucose, Gibco, Life Technologies, Carlsbad, CA, USA), supplemented with 10% autologous serum and 1X antibiotic/antimycotic solution (Gibco, Life Technologies, Carlsbad, CA, USA). The cells were then filtered through a 100µm filter (BD, Franklin Lakes, NJ, USA), and seeded at a density of 6 × 10^4^/cm^2^ in a Growth medium. After one week, floating cells were removed, and the remaining cells were cultured for 2–3 weeks until reaching a density of 0.5–1 × 10^7^ (second or third passage).

### 2.3. Safety Assessment and Cell Administration

Cells underwent testing for bacterial and mycoplasma sterility, analysis of expression of mesenchymal surface markers (CD73, CD90, and CD105), and hematopoietic surface markers (CD34 and CD45). Cell viability was >90%. Cells were resuspended in 1 mL of 1xPBS (Gibco, Life Technologies, USA), immediately transferred to the hospital, loaded into 2 mL sterile syringes, and injected into the affected joint within one hour of harvesting. No prior preparation or premedication of the patient was undertaken. No additional substances were injected into the knee joint. All patients were advised to abstain from using analgesics, anti-inflammatory drugs, immunosuppressants, or any form of physical therapy for one month before and six months following the stem cell application.

### 2.4. Scoring and Evaluation

Knees are clinically evaluated by the Hospital for Special Surgery Knee (HSS), Knee Society clinical rating system (KS), and Tegner–Lysholm (TL) scores. In addition, a Visual analog scale (VAS) was used for assessing the pain level, and knee range of motion (ROM) was measured.

To assess structural changes, two methods were used, both applied and evaluated by one person, a researcher who was blinded to the clinical scoring results: 

Kellgren–Lawrence (KL) classification was performed using knee roentgenograms both from baseline and from all follow-up intervals. This widely used X-ray-based grading system quantifies the following knee osteoarthritis landmarks: joint space narrowing and osteophyte formation, with grades ranging from 0 to 4, and has solid validity and sensitivity [24,25]. We opted for KL because of its compatibility with other knee osteoarthritis studies.

Magnetic Resonance Observation of Cartilage Repair Tissue (MOCART) score was performed using MRI images (T2 FSE sequence, 1–1.5 T) at baseline, 18 months, and 84 months. The scoring method is comprehensive and well-validated in the literature [26,27].

### 2.5. Statistical Analysis

The data were analyzed using online statistical tools available at Microsoft Excel 2016 (version 16.0.4266.1001) for parameter tests and calculators available from https://www.socscistatistics.com (accessed on 5 May 2024) for non-parameter tests. The data are reported as the mean (arithmetic mean ± standard deviation). Paired Student’s *t*-test was used for the interval, and the Wilcoxon signed-rank test was used for ordinal data differences between two repeated measures. The ANOVA with Bonferroni post hoc test was used for repeated measures. The normal distribution of data sets was determined by the Shapiro–Wilk test. All tests were two-sided and differences were considered to be significant when *p* < 0.05 and highly significant when *p* < 0.01 in all cases.

## 3. Results

Body mass index did not change significantly during the whole follow-up period: the baseline mean BMI was 29.5 kg/m^2^ ranging from 25–34 kg/m^2^, at 60 months BMI was 30.3 kg/m^2^ and after 84 months it was 29.2 kg/m^2^ ranging from 22.5–35.0 kg/m^2^ (Student *t*-test, *t* = −1.121127, *p* = 0.29126) (Table 1).

### 3.1. Clinical Results 

**Figure 1:**
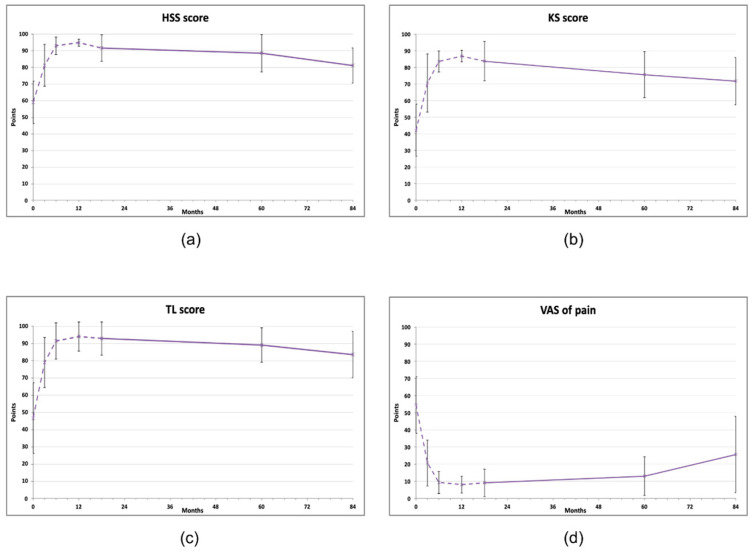
Clinical scores of knee joints treated with AMSCs (n = 11), assessed in indicated intervals (baseline, 3, 6, 12, 18, 60, 84 months). (**a**) Hospital for Special Surgery (HSS) knee score, (**b**) Knee society (KS) score, (**c**) Tegner–Lysholm score, (**d**) Visual analog scale (VAS) of pain. The dashed line represents the results published previously [18]. The vertical bars show the standard deviation.

At 60 months after the treatment, all clinical scores for knees were significantly improved compared to baseline levels, but at 84 months, all of them showed a declining trend (repeated measures ANOVA, Figure 1, Table 2). The KS score improved from initial 42.1 ± 15.71 to 75.6 ± 13.88 at 60 months (*p* < 0.05) and then decreased to 69.7 ± 15.64 (*p* > 0.05). A similar pattern was observed for the HSS score (baseline 59.0 ± 12.68; 60 months: 88.5 ± 11.25, *p* < 0.01 and 80 months: 79.8 ± 11.36, *p* > 0.05) and in the TL score (baseline 46.7 ± 20.5; 60 months: 89.1 ± 9.94, *p* < 0.01 and 80 months: 81.1± 15.66, *p* < 0.05) (Figure 1b, c). At 84 months, only the TL score was still statistically significantly improved compared to the baseline value (81.1 ± 15.66, *p* < 0.05). All measurements are available in Supplementary Material, Table S1.

**Figure 2:**
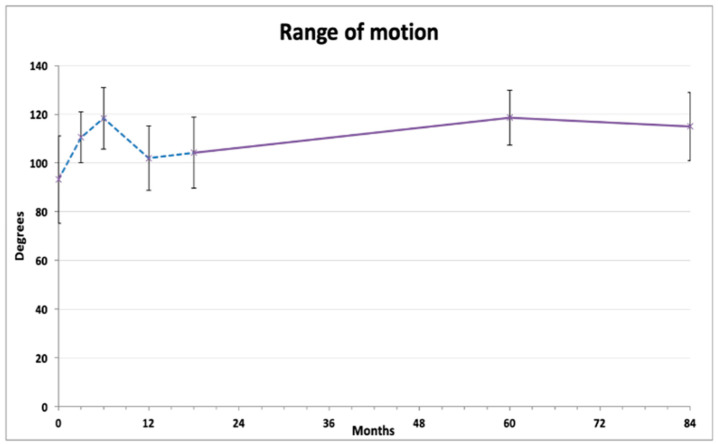
Average range of knee flexion (degrees). The dashed line represents the results published previously [18].

Knee range of motion exhibited a tidal pattern (Figure 2). After initial gradual improvement (at 3 and 6 months), the ROM values decreased 12 months after injection. On further measurements, a steady and lasting improvement was observed compared to baseline, reaching 118.6 ± 11.28 degrees at 60 months and 115.5 ± 13.94 degrees at 84 months (both *p* < 0.01). Repeated measures ANOVA with Bonferroni post hoc correction revealed that 6 months and 60 months results were significantly better than the baseline average, and that the 12 months and 18 months results were significantly lower than both 6 months and 60 months (Table 2).

### 3.2. Radiological Results

#### 3.2.1. X-ray Examination

**Figure 3:**
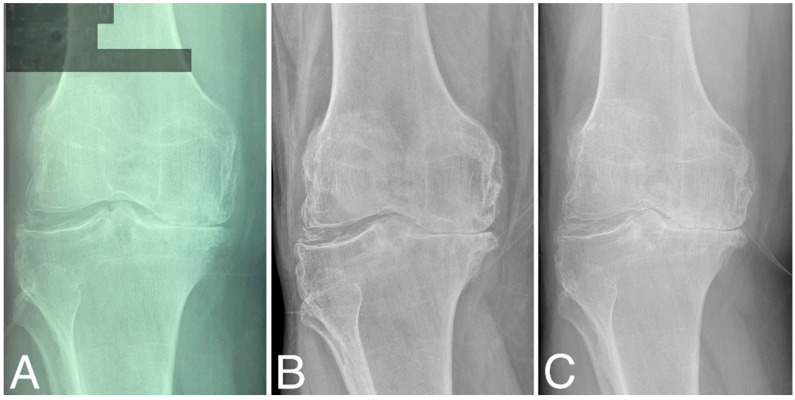
X-ray images of the right knee (**A**) at baseline, (**B**) at 60 months time point, and (**C**) at 84 months time point; all are classified as KL grade 4.

Structural changes in the distal femur and proximal tibia are evaluated according to KL classification (Table 1). At baseline, three patients were classified as grade 2, four as grade 3, and four as grade 4. At 60 months, KL scores did not change significantly (two as grade 2, three as grade 3, and five as grade 4), (Wilcoxon signed-rank test, Z = −1.095445, *p* = 0.273322). At 80 months, KL grades decreased significantly compared to that at baseline (three as grade 3 and seven as grade 4), (Wilcoxon signed-rank test, Z = −2.201398, *p* = 0.027708). Representative radiographies are given in Figure 3.

#### 3.2.2. Magnetic Resonance Examination

**Figure 4:**
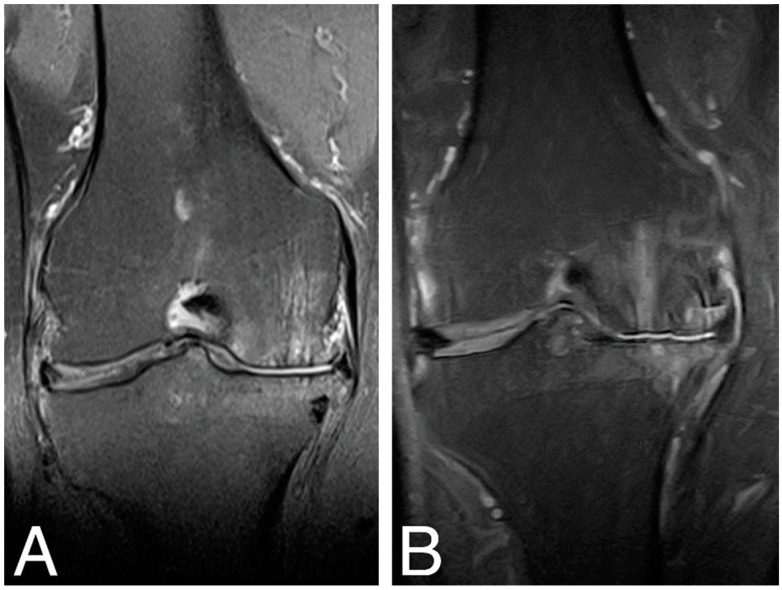
MRI images of the right knee, T2 sequence, and mid-joint frontal plane. (**A**) baseline (MOCART score 0.4); (**B**) 84 months time point (MOCART score 0.35).

MRI examination of the treated knee joints was performed at baseline, 18 months [18], and 84 months time points. Results were ambiguous, showing both an increase in subchondral bone edema and consolidation of the articular cartilaginous surface. Figure 4. shows MRI findings of the same patient as in Figure 3.

**Figure 5:**
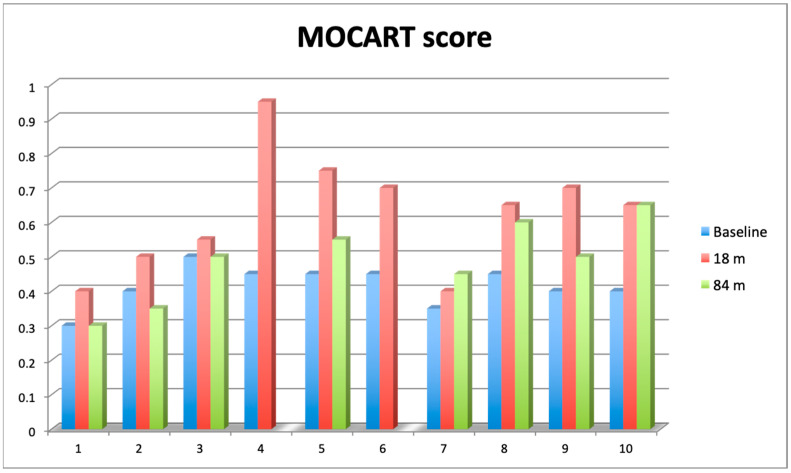
Comparison of 2D Magnetic Resonance Observation of Cartilage Repair Tissue (MOCART) score assessed at baseline (blue bars), after 18 months (red bars) and 84 months (green bars). Value of the score ranges from 0 (complete destruction of articular cartilage) to 1 (intact articular cartilage).

Knee articular cartilage structure and integrity were evaluated using the MOCART score (range from 0–1) (Figure 5), where a higher score indicates better cartilage structure. The average MOCART score at 84 months was 0.49 ± 0.119, which is significantly worse than 0.63 ± 0.172 observed at 18 months (Wilcoxon signed-rank test, Z = −2.028370, *p* = 0.042522), but still significantly better than the baseline value (0.42 ± 0.072, Z = −1.991741, *p* = 0.046399). In two cases, we observed clear cartilage deterioration with scores at 84 mo decreased back to baseline values (0.3 and 0.4). Conversely, in another two cases, the 84 months score was either equal to (0.65) or even higher (0.45) than the 18 months score. The four other cases showed some degree of MOCART score lowering between 18 months and 84 months but still above baseline values (Supplementary Material, Table S2).

## 4. Discussion

Many studies and clinical trials have shown beneficial effects of the stem cell therapy for the treatment of cartilage defects [2,15,16,17,20,21]. No severe adverse effects were shown for a single injection, and all patients tolerated the applied therapy well [28]. The use of naïve stem cells, injected without carriers or scaffolds, is a minimally invasive and safe treatment option. There are several critical steps in this procedure, from cell sterility and maintenance of cell integrity to crucial prevention of cell mutagenesis, which is why prolonged passaging should be avoided. When a source of stem cells for the treatment of OA is considered, there are many advantages of adipose-tissue-derived MSCs over bone-marrow-derived MSCs (BMMSCs), which are mostly considered for the treatment of OA [29]. AMSCs do not decrease their differentiation capacity nor osteogenesis and clonogenicity with age or toward the chondrocyte lineage, which is crucial for this age-related disease [30,31]. They exhibit a regenerative profile superior to that of BMMSCs, and display greater resistance to hypoxia-induced apoptosis and higher telomerase activity [30]. 

Several studies have shown that clinical and radiological improvements do not correlate with the number of cells used. These studies showed that lower doses have beneficial effects, especially for pain relief and range of motion [2,15,16,17,18,19]. A recent meta-analysis showed that lower doses can be significantly powerful in the treatment of OA [32]. Studies that used high-dose MSCs showed superior effects on cartilage thickness [20,21,22,23,33]. So far, two studies examined the use of multiple injections of AMSCs for the treatment of OA [22,33]. In a study by Song et al. [22], patients were first treated with two injections of low-, mid-, and high-dose stem cells at baseline and 3 weeks after liposuction. Forty-eight weeks later, they received a third injection of a high dose (0.5 × 10^8^ cells per joint). The outcomes were measured at 48, 72, and 96 weeks after liposuction. Although this study confirmed that high doses have an impact on cartilage thickness, they showed that lower doses have fast, significant, and persistent beneficial effects that are measured clinically. In a study by Freitag et al. [33], two groups of patients were treated with 1 × 10^8^ cells per joint: one group received a single dose and the other group received two doses within a 6-month interval. The outcomes were measured 3, 6, and 12 months after the first injection. Results showed good tolerance of the second injection and comparable clinical outcomes in both treated groups. In the second group, cartilage thickness was improved as well. There was a third treatment group in this study planned to receive five injections of 0.4 × 10^8^ AMSCs at baseline, 1, 2, 3, and 6 months, but this group was revoked due to severe adverse effects. This result is of great importance and should be taken into account when multiple injections of stem cells are considered. From our point of view, the lowest dose that is effective should be considered for usage in clinical praxis, mostly because of the high costs of stem cell preparation and the possible need for repeated injections. That was one of the rationales for choosing the dose 0.5–1 × 10^7^ cells used in our study. Another is that autologous serum was used for cell isolation and cultivation, which is a technical limitation for growing larger numbers of cells.

In our previous paper [18], a dose of AMSCs (0.5–1 × 10^7^) was administered and we documented that treatment results remain statistically improved 18 months after stem cell injection, regardless of the degree of the cartilage damage and the dose of applied cells. Here, we showed that at 60 months, except for one patient, functional improvement was still significant, as was quality of life, including a return to standard everyday activities, walking without mobility aids, and even modest recreational activities. All clinical scores as well as the VAS of pain were still significantly improved compared to those at baseline. At 84 months, the clinical scores decreased significantly, but the structural characteristics, measured by MRI, remained significantly better than those measured at baseline.

To our knowledge, our study has the longest follow-up period reported in the literature so far. Davatchi et al. reported 5 years follow-up of three patients treated with 8–9 × 10^6^ BMMSCs [34]. Similar to our study, their patients had moderate to severe knee OA and their results showed that at a 5-year time point, the parameters decreased compared to those at the 6 months, but were still better compared to those at baseline. It is interesting to note that although the improvement started to decrease during follow-up, the transplanted knee joint declined slower than the contralateral non-treated knee (which was in better condition at baseline). 

The use of autologous serum (AS) for the cultivation of MSCs, as performed in our study, could be a better choice compared to the use of FBS, because cells grown in AS were shown to exhibit superior proliferative effects while maintaining the capacity for differentiation [35]. It was also shown that the AMSCs cultivated in AS maintain a mesenchymal phenotype and they retain the potential for multilineage differentiation [36]. In addition to reducing the risk of xenogeneic immune responses resulting from animal proteins in FBS, autologous serum possibly offers superior results compared to cells cultured in FBS. These findings support the argument for the use of autologous serum instead of FBS for cell growth for therapeutic applications.

Our research faces several constraints. The primary limitation is the absence of control subjects for comparative analysis of the findings. The inclusion of a control group receiving alternative treatment, such as HA or other lubricants, was not feasible during the study’s preparation phase due to hospital policies. Another limitation arises from the small cohort of patients included in the study, significantly affecting statistical analysis. This is associated with budget constraints, as well as patients’ distrust in undergoing a treatment that was new and insufficiently explored at the time of the study design. In addition, different grades of knee OA, ranging from 2–4, made it impossible to assess the effect of stem cells on different levels of joint damage. Nonetheless, we consider it noteworthy that we tracked a cohort of patients over seven years, contributing significantly to the study of the therapeutic impact of mesenchymal stem cells in osteoarthritis treatment, showing good long-term tolerance and long-lasting effects of the therapy.

## 5. Conclusions

The favorable tolerance and long-term improvement in therapy outcomes, as demonstrated in our study, indicate that stem cell therapy is a safe and effective approach for treating OA. Our findings suggest that the effects of a single dose of 0.5–1 × 10^7^ stem cells last for 60 months from baseline, with a noticeable decline in clinical outcomes observed at the 7-year time point.

## Figures and Tables

**Table 1:** Characteristics of OA patients.

	Baseline	60 months	84 months
Age (year), mean (SD)	63 (10.4)	69.3 years (44–83)	72.2 years (46.3–85.5)
Sex (No)	3 male, 6 female	2 male, 6 female	2 male, 6 female
BMI, mean (SD) (kg/m^2^)	29.5 (3.97)	30.4 (4.79)	29.2 (4.03)
KL grade 1	0	0	0
KL grade 2	4	2	0
KL grade 3	3	3	3
KL grade 4	4	5	7

BMI (Body-mass index) = body weight/(body height)^2^. KL (Kellgren-Lawrence) score.

**Table 2:** Clinical results of the treatment with ADMCSs during the 84 months follow-up.

	Baseline	3 months	6 months	12 months	18 months	60 months	84 months	ANOVA
KS score (max = 100)	42.1 ± 15.71	70.6 ± 17.53 **	83.5 ± 6.36 **	86.8 ± 3.49 **	83.7 ± 11.86 **	75.6 ± 13.88 *	69.7 ± 15.64	F = 14.94 **
HSS score (max = 100)	59.0 ± 12.68	81.2 ± 12.64 **	92.9 ± 5.26	94.8 ± 2.09 **	91.6 ± 7.93 **	88.5 ± 11.25 **	79.8 ± 11.36	F = 27.66 **
TL score (max = 100)	46.7 ± 20.5	79.0 ± 14.56 **	91.5 ± 10.55 **	94.1 ± 8.42 **	92.9 ± 9.55 **	89.1 ± 9.94 **	81.1 ± 15.66 *	F = 30.01 **
VAS of pain (max = 100)	54.5 ± 16.5	20.7 ± 13.3 **	9.3 ± 6.5 **	8.0 ± 4.9 **	9.1 ± 7.9 **	13.0 ± 1.14 **	28.7 ± 24.39	F = 15.08 **
ROM (degrees)	93.2 ± 17.93	110.5 ± 10.42 **	118.3 ± 12.69 **	99.2 ± 10.29	102.4 ± 13.88	118.6 ± 11.28 **	115.5 ± 13.94 **	F = 7.03 **

** *p* < 0.01; * *p* < 0.05 (repeated measurements ANOVA with Bonferroni post-hoc test, compared to baseline values).

## Data Availability

The original contributions presented in the study are included in the Supplementary Material, further inquiries can be directed to the corresponding author.

## Supplementary Materials

The following supporting information can be downloaded at: https://www.mdpi.com/article/10.3390/jcm13133861/s1, Table S1. Clinical measurements. Table S2. MRI 2D MOCART score.
