# Supplementary material for: Seven-Year Longitudinal Study: Clinical Evaluation of Knee Osteoarthritic Patients Treated with Mesenchymal Stem Cells
Source: J Clin Med. 2024 Jun 30;13(13):3861. doi: 10.3390/jcm13133861 (PMC11242523; doi:10.3390/jcm13133861)
Supplement: Supplementary file 1 [file jcm-13-03861-s001.zip › jcm-3067540-supplementary.pdf]

Supplementary material

Seven-Year Longitudinal Study: Clinical Evaluation of Knee Osteoarthritic Patients Treated with Mesenchymal Stem Cells

Table S1 . Clinical measurements

	Patients	P1	P2	P3	P4	P5	P6	P7	P8	P9	P10	Shapiro W	p value
ROM	Baseline	106	90	110	90	100	85	90	51	80	112	0.893053	0.1835
	60m	124	95	115	122	127	110	121	130	110	132		
	84m	105	100	135	120	110	100	100	125	120	135		
HSS	Baseline	78	64	58	47	70	42	64	45	44	64	0.910585	0.2850
	60m	92	78	98	97	97	79	69	98	78	99		
	84m	86	74	98	86	68	81	70	97	73	78		
KS	Baseline	58	33	33	29	57	47	61	9	47	36	0.931912	0.4669
	60m	70	51	86	85	85	73	69	91	57	89		
	84m	73	51	86	80	53	71	70	90	57	86		
TL	Baseline	83	64	31	18	46	34	54	21	41	51	0.96747	0.8664
	60m	94	89	95	90	90	67	89	99	78	100		
	84m	92	92	94	74	57	83	70	99	80	95		
VAS	Baseline	1.9	5.9	7.1	6.7	5.1	5.5	4.5	8.2	4.2	5.5	0.969067	0.8821
	60m	0.7	0.9	1.1	0.7	0.4	2.2	4	0.5	2	0.5		
	84m	1.6	3.4	1.6	1.7	8.5	2.6	2.2	2.3	0.9	0.8		

ROM – Range of motion of the knee joint (degrees)
HSS – Hospital for Special Surgery score (max = 100 points for healthy joint)
KS – Knee Society score (max = 100 points for healthy joint)
TL – Tegner-Lysholm score (max = 100 points for healthy joint)
VAS – Visual analog scale of pain ((max = 10 points if no presence of pain)

Table S2. MRI 2D MOCART score

	Patients	P1	P2	P3	P4	P5	P6	P7	P8	P9	P10
Baseline	degree of cartilage repair	10	5	10	20	5	5	5	5	5	10
	Integration to border zone	10	10	10	10	10	10	10	15	10	10
	surface	5	5	5	5	5	5	5	5	5	5
	Adhesions	5	5	5	5	5	5	5	5	5	5
	Structure	0	0	5	0	0	0	0	0	5	0
	signal intensity	0	10	10	0	10	10	10	10	10	0
	subch lamina	0	5	5	0	5	5	0	0	0	5
	subch bone	0	0	0	0	5	5	0	5	0	5
	effusion	0	0	0	5	0	0	0	0	0	0
	MOCART score	0.3	0.4	0.5	0.45	0.45	0.45	0.35	0.45	0.4	0.4
18m	degree of cartilage repair	10	10	15	20	10	10	10	15	10	10
	Integration to border zone	5	5	10	15	10	15	10	15	15	15
	surface rt	5	5	5	10	5	10	5	5	10	10
	Adhesions	5	5	5	5	5	5	5	5	5	5
	Structure	0	5	5	5	5	5	0	5	5	5
	signal intensity	10	10	10	30	30	10	10	10	10	10
	subch lamina	0	5	0	5	5	5	0	5	5	5
	subch bone	5	0	0	0	0	5	0	0	5	5
	effusion	0	5	5	5	5	5	0	5	5	0
	MOCART score	0.4	0.5	0.55	0.95	0.75	0.7	0.4	0.65	0.7	0.65
84m	degree of cartilage repair	10	10	15		10		10	10	10	15
	Integration to border zone	5	5	5		10		10	15	10	10
	surface rt	5	5	5		5		5	10	10	5
	Adhesions	5	5	5		5		5	5	5	5
	Structure	0	5	5		5		0	5	0	5
	signal intensity	10	10	10		10		10	10	10	10
	subch lamina	0	0	0		0		0	0	0	5
	subch bone	0	0	0		5		0	0	0	5
	effusion	5	5	5		5		5	5	5	5
	MOCART score	0.3	0.35	0.5		0.55		0.45	0.6	0.5	0.65
